# Mefloquine pharmacokinetics and mefloquine-artesunate effectiveness in Peruvian patients with uncomplicated *Plasmodium falciparum *malaria

**DOI:** 10.1186/1475-2875-8-58

**Published:** 2009-04-09

**Authors:** Julie Gutman, Michael Green, Salomon Durand, Ofelia Villalva Rojas, Babita Ganguly, Wilmer Marquiño Quezada, Gregory C Utz, Laurence Slutsker, Trenton K Ruebush, David J Bacon

**Affiliations:** 1 Division of Parasitic Diseases, Centers for Disease Control and Prevention (CDC), Atlanta, GA USA; 2 Emory University School of Medicine, Department of Pediatric Infectious Disease, Atlanta, GA 30322, USA; 3 U.S. Naval Medical Research Center Detachment (NMRCD), Iquitos, Peru; 4 Instituto Nacional de Salud, Lima, Peru

## Abstract

**Background:**

Artemisinin-based combination therapy (ACT) is recommended as a means of prolonging the effectiveness of first-line malaria treatment regimens. Different brands of mefloquine (MQ) have been reported to be non-bioequivalent; this could result in sub-therapeutic levels of mefloquine with decreased efficacy. In 2002, mefloquine-artesunate (MQ-AS) combination therapy was adopted as the first-line treatment for uncomplicated *Plasmodium falciparum *malaria in the Amazon region of Peru. Although MQ resistance has yet to be reported from the Peruvian Amazon, it has been reported from other countries in the Amazon Region. Therefore, continuous monitoring is warranted to ensure that the first-line therapy remains efficacious. This study examines the *in vivo *efficacy and pharmacokinetic parameters through Day 56 of three commercial formulations of MQ (Lariam^®^, Mephaquin^®^, and Mefloquina-AC^® ^Farma) given in combination with artesunate.

**Methods:**

Thirty-nine non-pregnant adults with *P. falciparum *mono-infection were randomly assigned to receive artesunate in combination with either (1) Lariam, (2) Mephaquin, or (3) Mefloquina AC. Patients were assessed on Day 0 (with blood samples for pharmacokinetics at 0, 2, 4, and 8 hours), 1, 2, 3, 7, and then weekly until day 56. Clinical and parasitological outcomes were based on the standardized WHO protocol.

Whole blood mefloquine concentrations were determined by high-performance liquid chromatography and pharmacokinetic parameters were determined using non-compartmental analysis of concentration versus time data.

**Results:**

By day 3, all patients had cleared parasitaemia except for one patient in the AC Farma arm; this patient cleared by day 4. No recurrences of parasitaemia were seen in any of the 34 patients. All three MQ formulations had a terminal half-life of 14–15 days and time to maximum plasma concentration of 45–52 hours. The maximal concentration (C_max_) and interquartile range was 2,820 ng/ml (2,614–3,108) for Lariam, 2,500 ng/ml (2,363–2,713) for Mephaquin, and 2,750 ng/ml (2,550–3,000) for Mefloquina AC Farma. The pharmacokinetics of the three formulations were generally similar, with the exception of the C_max _of Mephaquin which was significantly different to that of Lariam (*p *= 0.04).

**Conclusion:**

All three formulations had similar pharmacokinetics; in addition, the pharmacokinetics seen in this Peruvian population were similar to reports from other ethnic groups. All patients rapidly cleared their parasitaemia with no evidence of recrudescence by Day 56. Continued surveillance is needed to ensure that patients continue to receive optimal therapy.

## Background

The World Health Organization recommends the use of artemisinin-based combination therapy (ACT) as a means of prolonging the effectiveness of first-line malarial treatment regimens [1-3]. The effectiveness of ACT relies on the different modes of action from the partnered drugs. The artemisinin derivatives, (e.g. artesunate, artemether, dihydroartemisinin) in the ACT are eliminated rapidly, while the partner drugs (e.g. mefloquine, amodiaquine, lumefantrine, piperaquine) are eliminated more slowly. The artemisinin derivative and its partner drug each protect against selection for resistance against the other drug [3]. Therefore, it is important that the non-artemisinin partner drug maintains consistent pharmacokinetic and pharmacodynamic properties.

There have been reports of non-bioequivalence of mefloquine (MQ) tablets produced from different companies. For example, in a randomized cross-over study of healthy volunteers, Weidekamm *et al *showed a significant difference in the bioequivalence of Mephaquin^® ^(Mepha) compared with the reference product, Lariam^® ^(Roche), with Lariam having nearly twice the maximum plasma concentration of Mephaquin (C_max _1018 ng/ml vs. 656 ng/ml respectively) and requiring approximately 1/3 of the time to reach maximum concentration (t_max _13 vs 46 h) when pharmacokinetic parameters were assessed using plasma levels of MQ [4]. A study of whole blood MQ levels in patients with acute uncomplicated falciparum malaria showed that the relative bioavailability of Eloquine^® ^(Medochemie Ltd., Cyprus) was only 72% and Mephaquin only 49% of that of Lariam following administration of dihydroartemisinin [5]. It is unclear whether this is clinically relevant [6,7].

In 2002, mefloquine-artesunate (MQ-AS) combination therapy was adopted as the first-line treatment for uncomplicated *Plasmodium falciparum *malaria in the Amazon region of Peru due to increasing failures after treatment with chloroquine or sulphadoxine-pyrimethamine [8]. Studies done prior to the introduction of MQ-AS in Peru and other countries in Latin America showed that both MQ and MQ-AS were highly efficacious; however, these studies followed patients for only 28 days, as per the WHO protocol at that time [8-13]. A 28-day follow-up could underestimate the true failure rate by as much as 40% [14]. Although, MQ resistance has yet to be reported from the Peruvian Amazon[15], it has been reported from other countries in the Amazon Region [16-18]. Therefore, continuous monitoring is warranted to ensure that the first-line therapy remains efficacious.

Since adoption of MQ-AS as the first-line treatment, MQ from AC Farma and Mepha have been used routinely in Peru. The significantly lower bioavailability of Mephaquine reported by the previous study raised concern that the recommended dose of 25 mg/kg might be insufficient to achieve therapeutic MQ blood levels in all patients in Peru. Therefore, the *in vivo *efficacy and pharmacokinetic parameters through Day 56 of three commercial tablet formulations of MQ were compared: Lariam, Mephaquin, and Mefloquine-(AC Farma), when given in combination with artesunate.

## Methods

### Patients

The study was conducted between March 2004 and February 2005 at the Hospital de Apoyo, located in the city of Iquitos, in the Peruvian Amazon region. Patients were enrolled in five health centers located in the outskirts of Iquitos. The inclusion criteria for enrolling patients included: male or non-pregnant female ≥ 18 years of age, mono-infection with *P. falciparum*, with a parasite density between 250 and 50,000 asexual parasites/mm^3 ^as determined by microscopic examination of a thick blood smear, fever or history of fever in the previous 48 hours, informed consent from patient, and a willingness to be hospitalized for the first 24 hours after initiation of therapy and to return for follow-up visits through day 56. Patients exhibiting evidence of severe malaria, a history of an underlying chronic disease or illness that could interfere with the absorption of MQ, a history of hypersensitivity to MQ, or a history of neuropsychiatric illness or cardiac conduction problems were excluded.

Eligible patients were assigned to one of three treatment arms using a list of randomly generated numbers; (1) Lariam (Hoffman-LaRoche Ltd, Basel, Switzerland; 250 mg/tablet) used as the reference, (2) Mephaquin (Mepha S.A. Aesch-Basel, Switzerland; 250 mg/tablet), and (3) Mefloquina AC (AC Farma Laboratories S.A. Lima, Peru; 250 mg/tablet). All drugs were administered as whole tablets with water at a dose of 25 mg/kg (15 mg/kg on the first day and 10 mg/kg on the second day) along with artesunate at a dose of 4 mg/kg/day for three days under supervision by clinical staff. No additional food or drink was given until 2 hours following the administration. Patients were closely observed for 60 minutes after treatment for adverse reactions or vomiting. Any patients who vomited within 60 minutes of the dose were re-dosed with a full dose, but were excluded from the study. No food was allowed for two hours after drug administration.

### Clinical and laboratory assessments

Patients were assessed on Day 0, 1, 2, 3, 7, and then weekly until day 56. At each visit patients were questioned regarding possible adverse effects to mefloquine and artesunate.

### Evaluation of in vivo efficacy

Blood smears were taken by finger prick every day until negative for parasitaemia and then on days 7, 14, 21, 28, 35, 42, and 56. Thick blood smears were prepared with Giemsa stain and the number of parasites per 200 WBC counted. Parasite density was estimated assuming an average WBC count of 6,000/mm^3^.

Clinical and parasitological outcomes were based on the standardized WHO protocol [19]. The adjusted Wald test was used to calculate the 95% confidence interval [20].

### Evaluation of mefloquine pharmacokinetics

Three ml of whole blood for MQ analysis was taken by venopuncture before administration of the first dose of MQ and then at 2, 4, 8, 24, 48, and 72 hours and 7, 14, 21, 28, 35, 42, and 56 days. The number of early sampling times was minimized due to logistical constraints and to ease patient discomfort. Samples were stored at -20°C prior to analysis and all samples were analysed within 21 months of collection. MQ concentrations in whole blood were determined by high-performance liquid chromatography using a modified procedure described by Green *et al *[21]. The modification involved the use of a 150 × 4.6 mm octadecylsilica column with a mobile phase consisting of 30% acetonitrile and 70% 0.05 M phosphate buffer (pH = 3) and a flow rate of 1.5 ml/min. Interassay variability was assessed from 15 standard curve runs of whole blood spiked with MQ concentrations of 250, 500, 1000, and 2500 ng/ml. Interassay precision (expressed as coefficient of variation [CV]) was 23%, 20%, 15%, and 2%, respectively while interassay accuracy (CV) was 2%, 4%, -4%, and 0%, respectively.

Pharmacokinetic parameters for MQ were determined using non-compartmental analysis of the whole blood concentration versus time data [22]. The points were plotted and the area-under-the-curve (AUC_0→t_) from time of administration to the time of the last quantifiable measurement (C_t_) was calculated directly from the log concentration versus time plot using the trapezoidal rule [23]. The AUC from 0 to infinity (AUC_∟_) was determined by combining the AUC_0→t _with the area extrapolated from the concentration curve as determined from the concentration of the last quantifiable time divided by the elimination constant (AUC_∟ _= AUC_0→t _+ C/k_el_). The last quantifiable concentration occurred at 1008 hours for most subjects (>90% for each arm). The slope of the least squares regression analysis from the last four log concentration-time points was used to determine the terminal elimination constant (k_el_) and half life (t_1/2 _= ln2/k_el_). The time at which maximum concentration occurred (t_max_), and the maximum concentration (C_max_) were obtained directly from fitting the concentration versus time data to a triexponential model (y = ae^-bx ^+ ce^-dx ^+ ge^-hx^) using Scientist™ vers.2.01 curve fitting programme [24]. C_max _and t_max _were determined from the curve maximum from each plot, therefore no equations were used beside the triexponential. The parameters were determined from the data sets collected from individual patients.

### Statistical analysis

Statistical analysis was done using SAS Version 9.1.3 (Cary, NC). Fisher's Exact test was used for comparison of group parameters due to the small group size. The parasite densities showed a highly skewed distribution; therefore, they were log-transformed and the Wilcoxon Rank Sum test was used to test for differences between the densities after log-transformation. Results were considered statistically significant with a p-value < 0.05. Based on previous studies showing that Mephaquin has an AUC and C_max _approximately one-half that of Lariam, 33 adult patients, 11 of whom are treated with Lariam, 11 with Mephaquin, and 11 with mefloquine (AC Farma) would be sufficient to detect a significant difference between the AUC and C_max _for each of the two formulations and Lariam at a 5% level of significance and a power of 80%. Since biological parameters usually do not exhibit a normal distribution [25], the Mann-Whitney test was used to observe differences between the test groups relative to the reference group

### Ethics

The study protocol was approved by the institutional review boards of the US Navy Medical Research Center, Lima, Peru, Instituto Nacional de Salud del Peru, Lima, Peru and the Centers for Disease Control and Prevention, Atlanta, GA, USA. Patients were informed about the study and enrolled voluntarily after having read and signed the informed consent document. This study was registered at ClinicalTrials.gov, number NCT00544024.

## Results

### Clinical response

Thirty-nine adult subjects ranging in age from 18–61 years (mean 36 years) were initially enrolled in the study. Seventy-two percent of the patients were male. Each arm initially consisted of 13 individuals. A total of 5 subjects did not complete the full follow-up period. One subject was excluded from the Mephaquin arm on day 1 for hypertension requiring medication. Two were withdrawn due to adverse events: one from the Lariam arm on day 5, and one from the AC Farma arm on D28. Two were lost to follow-up: one from the AC Farma arm on day 35 and one from the Lariam arm on day 37. Thus, 11, 12, and 11 patients remained for full analysis of efficacy in the Lariam, Mephaquin, and AC Farma arms, respectively. The three groups were similar with regard to gender, age, and weight (Table 1). All patients had a history of fever in the previous 48 hours; 21% of patients had a documented fever (axillary temperature ≥ 37.5°C) at enrollment. Parasite density (parasites/mm^3^) on admission was similar for each treatment arm [(geometric mean (range)]: Lariam = 8,159 (3,085, 21,578), AC Farma = 4,280 (1,515, 12,088), Mephaquin = 9,224 (3,450, 24,660). There were no significant differences in the rate of parasite clearance between treatment arms (Table 2). By day 3, all patients had cleared parasitaemia except for one patient in the AC Farma arm; this patient cleared by day 4. No recurrences of parasitaemia were seen in any of the 34 patients; drug efficacy was 100%, 95% confidence interval 87.6–100% for the three arms combined, 69%–100% for Lariam and AC Farma, and 71%–100% for Mephaquin.

**Table 1:** Characteristics of enrolled patients by treatment group

	**AC farma**	**Lariam**	**Mephaquin**
**N**	**13**	**13**	**13**

**Male N (%)**	8 (61.5)	9 (69)	11 (84.6)

**Age (years)**	33.6	35.4	39.5

**Fever at enrollment**	2 (15%)	3 (23%)	3 (23%)

**Days of fever**	4.8	5.2	3.7

**Geometric Mean Parasite density D0**	4279.97	8159.33	9224.15

**Table 2:** The daily geometric mean parasite density and 95% confidence interval for each treatment group is shown

**Parasite density**	**Lariam**	**Mephaquin**	**Mefloquina AC**	**p-value**
**D0**	8159 (3085–21,578)	9224 (3450–24,660)	4280 (1515–12,088)	0.51

**D1**	40 (7–212)	15 (3–65)	19 (2–131)	0.54

**D2**	0.38 (0–1.8)	0.48 (0–1.7)	0.88 (0–4.2)	0.86

**D3**	0	0	0.22 (0–0.87)	0.38

**D7**	0	0	0	1

No statistically significant difference was observed using the Wilcoxon Rank Sum test.

### Adverse events

One patient developed insomnia, anxiety and paranoia after receiving the second dose of Lariam; the drug was stopped and all symptoms resolved within 24–48 hours. The symptoms were attributed to mefloquine. No other drug related adverse events were reported. One patient in the AC Farma arm was withdrawn (Day 28) following development of meningoencephalitis, presumed to be viral. Two other patients in the AC Farma arm had adverse events deemed unrelated to the study drug and remained in the study; one developed a subarachnoid haemorrhage and was hospitalized on study Day 48, the other required surgery for a traumatic spleen injury on study day 14.

### Pharmacokinetics

**Figure 1:**
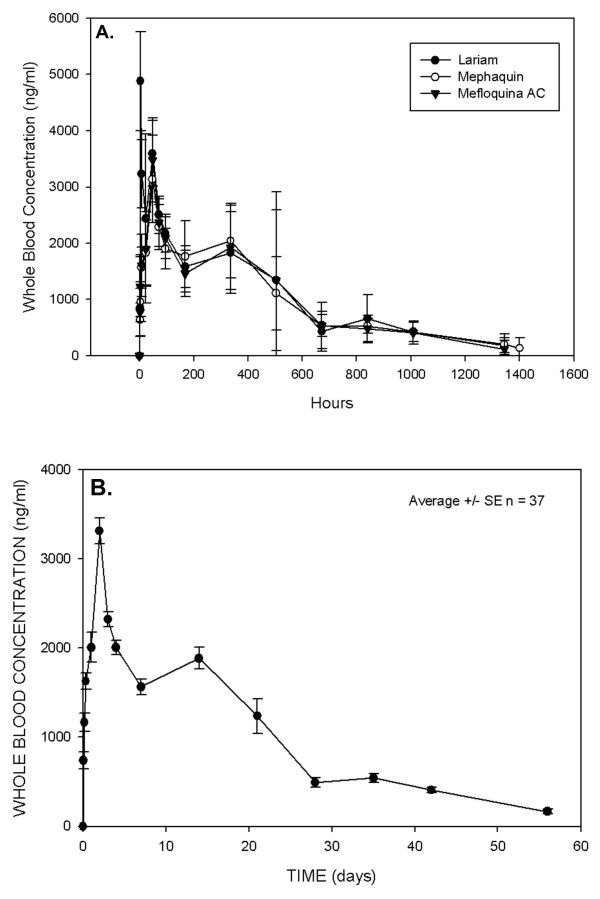
**Median whole-blood concentration-time profiles for mefloquine**. Mefloquine was administered in a total dose of 25 mg/kg (15 mg/kg on the first day and 10 mg/kg on the second day) in conjunction with artesunate given at 4 mg/kg/day for three days. Figure 1A. shows the whole-blood concentration-time profiles for each formulation separately, while Figure 1B shows the curve when all data points were combined. As can be seen, there were no significant differences between the formulations.

The pharmacokinetic analysis included all patients who had remained in the study until at least day 28, and thus excluded only two patients, one in the Mephaquin arm and one in the Lariam arm. Figure 1 illustrates the time versus median whole blood concentrations for the three tablet formulations. Table 3 shows the pharmacokinetic parameters for MQ following the administration of Lariam (reference), Mephaquin, and mefloquine-AC Farma in combination with artesunate to patients diagnosed with malaria. As the three formulations were not significantly different from one another, the overall pharmacokinetic parameters were also calculated. The results are presented as the median with the interquartile range. All three MQ formulations had a terminal half-life (t_1/2_) of 14.5–15 days and time to maximum plasma concentration (t_max_) of 45–52 hours. The maximal concentration (C_max_), expressed as ng/ml was 2,820 (2,614–3,108) for Lariam, 2,500 (2,363–2,713) for Mephaquin, and 2,750 (2,550–3,000) for Mefloquina AC Farma. The non-parametric Mann-Whitney test revealed a significant difference in the C_max _of Mephaquin relative to the reference (*P *< 0.05). There were no significant differences (*P *> 0.05) for all other parameters between the test and the reference groups. The coefficient of variation ranged from 16–37% for C_max _and AUC values.

**Table 3:** Pharmacokinetic parameters for three different formulations of mefloquine when administered in combination with artesunate to patients diagnosed with malaria.

	**Lariam** **(n = 12)**	**Mephaquin** **(n = 12)**	**Mefloquina AC** **(n = 13)**	**All** **(n = 37)**
**C_max _(ng/ml)**	2820 (2614–3108)	2500 (2363–2713)*	2750 (2550–3000)	2700 (2500–3000)
**AUC_0→t _(mg/ml/day)**	59.7 (41–67)	56.1 (51–60)	50.6 (41–67)	56.9 (44–66)
**AUC_∟ _(mg/ml/day)**	63.59 (43–77)	62.32 (60–68)	59.42 (47–71)	62.37 (47–75)
**t_max _(hours)**	45 (41–58)	51.5 (38–66)	45 (36–54)	45 (36–57)
**t_1/2 _(hours)**	346 (315–368)	365 (342–449)	347 (289–365)	347 (315–408)
**V_d/f _(L/kg)**	7.87 (7.0–10.8)	9.03 (8.16–9.83)	8.34 (7.0–11.0)	8.57 (7.3–10.7)
**C_l/f _(L/h/kg)**	0.016 (0.014–0.024)	0.017 (0.016–0.018)	0.018 (0.015–0.022)	0.017 (0.013–0.022)

* Significantly different from Lariam (*p *= 0.04)

The results are presented as the median with the interquartile range. The non-parametric Mann-Whitney test revealed a significant difference in the C_max _of Mephaquin relative to Lariam (p < 0.05). There were no significant differences between the test and the reference groups for any other parameters.

## Discussion

In this study, it was shown that although there is some manufacturer dependent variation in the drug levels of MQ, this did not appear to affect clinical outcomes. All patients rapidly cleared their parasitaemias with no evidence of recrudescence by Day 56. There was also no difference in occurrence of drug-related side effects between groups; all formulations were well-tolerated.

All three MQ formulations had a terminal half life (t_1/2_) of 14–15 days and time to maximum plasma concentration (t_max_) of 45–52 hours. This is similar to previous reports in the literature [26]; bioequivalence studies for Lariam conducted by Weidekamm *et al *[4] and Na-Bangchang *et al *[5] reveal t_1/2 _values of 16 and 11.5 days while t_max _values were 46 and 12 hours, respectively. The t_max _values may have corresponded more closely with Weidekamm *et al *[4] because of a more similar dosing schedule. Using the Mann-Whitney test, there were no differences in values of AUC, AUC_0-t_, t_max_, and t_1/2_between the three products. Although a statistically significant difference (*P *< 0.05) was observed in the C_max _of Mephaquin relative to the reference (Lariam, Table 1), no clinically significant difference could be detected. This group of subjects tended to be older (median age = 40) than the other arms (Lariam median age = 32, Mefloquina AC Pharma median age = 30) and may account for the slower absorbance.

A large relative standard deviation (%RSD) was observed for the C_max_and AUCs, ranging from ~16 to 37% for all formulations. The study design may have contributed to the high variability since no crossover scheme was employed and no attempt was made to match patients for confounding factors, including age, sex, weight, and degree of parasitaemia. Other studies have also reported large inter-individual variability (% relative standard deviation = 28–83%) for MQ pharmacokinetic parameters [5,6,27]. Many drugs may inherently exhibit highly variable pharmacokinetic parameters, but are generally safe and efficacious if the therapeutic index is not too narrow. Midha *et al *discusses various approaches to deal with highly variable drugs [28]. These include the broadening of acceptance limits based on drug variability, on the caveat that the interval should be prospectively defined and justified.

Following introduction of a new anti-malarial drug, it is important to continue to monitor drug efficacy. Although many studies use 28-days of follow-up, this length of follow-up may be inadequate for detecting early development of resistance. When combinations of drugs with discordant half-lives, such as mefloquine-artesunate, are used, the patient, in effect, receives monotherapy with the longer lasting drug for the tail end of therapy. This may lead to recrudescence, which may occur more than 4 weeks after administration of drug [29]. It is crucial to monitor for late treatment failures due to recrudescence [30-32].

## Conclusion

The pharmacokinetics of three formulations of MQ administered in conjunction with artesunate to patients with uncomplicated malaria were similar to each other; in addition, it was shown that they are similar in the Peruvian population to that described previously in Southeast Asian populations. This is the first study to demonstrate the pharmacokinetics of mefloquine in a South American population. All three formulations appeared equally efficacious in non-pregnant adults, although the small sample size precludes a definitive evaluation of efficacy. Continued surveillance is needed to ensure that patients continue to receive optimal therapy.

## Disclaimer

The views expressed in this article are those of the authors and do not necessarily reflect the official policy of the Department of the Navy, Department of Defense, nor the US Government. Some of the authors are military service members. This work was prepared as part of our official duties. Title 17 U.S.C. § 105 provides that "Copyright protection under this title is not available for any work of the United States Government." Title 17 U.S.C. § 101 defines a U.S. Government work as a work prepared by a military service member or employee of the U.S. Government as part of that person's official duties. The study protocol was approved by the Naval Medical Research Center institutional review board in compliance with all applicable federal regulations governing the protection of human subjects.

## Competing interests

The authors declare that they have no competing interests.

## Authors' contributions

JG participated in analysis and interpretation of the data and drafted the manuscript. MG performed the pharmacokinetics studies, analysed and interpreted the results of the pharmacokinetics studies, and drafted the manuscript. SD was critical in enrolling patients and collecting clinical data and samples. OVR performed the quantification of the blood samples. BG performed the HPLC analysis of the samples to quantitate the concentration of the drug and assisted with the pharmacokinetic analysis and calculations. WMQ made substantial contributions to acquisition of data and samples. GCU made substantial contributions to the design and implementation of the study, secured funding for the protocol, assisted with sample collection, provided oversight of the study conduct, and participated in analysis and interpretation of data. LS participated in analysis and interpretation of the data and helped to draft the manuscript. TKR conceived the study, participated in the study design and revision of the manuscript. DJB oversaw the daily operation of the study and contributed to the manuscript. All authors read and approved the final manuscript.
